# Infective Endocarditis: Still a Deadly Disease

**DOI:** 10.36660/abc.20190809

**Published:** 2020-01

**Authors:** Cristiane da Cruz Lamas

**Affiliations:** 1 Coordenação de Ensino e Pesquisa, Instituto Nacional de Cardiologia, Rio de janeiro, RJ - Brazil; 2 Centro Hospitalar, Instituto Nacional de Infectologia Evandro Chagas, Fiocruz, Rio de Janeiro, RJ - Brazil; 3 Universidade do Grande Rio (UNIGRANRIO), Rio de Janeiro, RJ - Brazil

**Keywords:** Heart Failure/physiopathology, Endocarditis/complications, Endocarditis/surgery, Hospital Mortality, Comorbidity, Septic shock, Echocardiography/methods

The important issue of in-hospital mortality in infective endocarditis (IE) is discussed by Marques et al.^1^ In-hospital mortality in the International Collaboration in Endocarditis (ICE) cohort (2000-2005) was 18%,^2^ similar to the 17% in the large European cohort recently published,^3^ both unacceptably high, considering that most patients included were from developed countries and voluntary registries.

In the present article, in-hospital mortality was 42/134 (31.3%), higher than expected. The identified risk factors for in-hospital mortality were *Staphylococcus aureus* etiology, negative blood-cultures, evidence of valve obstruction in echocardiography, heart failure secondary to IE and septic shock. Cardiac surgery was protective for mortality. To me, the most important message is “surgery was protective for mortality”. This has been shown in several studies.^1-6^

Surgical treatment is required in approximately half of the patients with IE because of severe complications, of which heart failure (acute or acute on chronic) is the most frequent, occurring in 40-60%.^7^ It represents the most common indication for surgery in left- sided native valve IE. Surgery may need to be performed on an emergency (within 24 h) or urgent (within a few days, 7 days) basis, irrespective of the duration of antibiotic treatment, or maybe delayed 1 or 2 weeks of antibiotic treatment.^7^ Although it is not clear which is the best timing,^6,8^ surely before the onset of acute heart failure seems a good time.^9^

A systematic review and meta-analysis evaluated papers where early versus late surgical intervention or medical management for IE were done.^5^ The definition used for early valve surgery in this publication was performance of surgery at 20 days or less of diagnosis of IE or during initial hospitalization. All-cause mortality was mentioned in 21 studies, and in the group that had early surgery, it was significantly lower than in the group without early surgical intervention (OR 0.61, 95% CI 0.50 to 0.74, p < 0.001). Heterogeneity was high among the included studies. However, regarding in-hospital mortality, a total of 11 studies reported on it and there was no significant difference between the early surgery and conventional therapy groups.^5^

Wang et al.^8^ addressed the issue of timing of surgery in patients with definite, left-sided IE according to the modified Duke criteria who underwent cardiac surgery during the index hospitalization.^8^ This was a prospective cohort from the ICE -PLUS study and involved 485 patients who were operated during the same admission. Notably, cases of device-related IE were excluded from the analysis, as were hemorrhagic stroke before surgery, nosocomial IE, and surgery performance more than 60 days from admission. A multivariable logistic regression model was fit to calculate a propensity score (probability) for early surgical treatment. The median time to surgery was 7 days ([IQR] 2-15). Patients who underwent earlier surgery had a lower percentage of preexisting heart failure (before IE diagnosis) but a higher rate of acute heart failure; no difference in 6-month survival across the quartiles (Quartile 1, surgery day 0 or 1; Q2, day 2 to 6; Q3 day 7 to 15; Q4 more than 15 days) of surgical timing was found. The risk of 6-month mortality was highest for patients who underwent surgery within the initial 2 days after admission or transfer. The authors concluded that the routine use of very early surgery for any indication is not supported by current data.^8^

The EURO-ENDO study involved a prospective cohort of 3116 adult patients (2470 from Europe), years 2016 to 2018 with a diagnosis of probable or definite IE.^3^ Cardiac surgery was indicated in 2160 (69.3%) patients but finally performed in only 1596 (73.9%) of them. In-hospital death occurred in 532 (17.1%) patients and was more frequent in prosthetic valve IE.^9^ Independent predictors of mortality were Charlson index, creatinine > 2 mg/dL, congestive heart failure, vegetation length > 10 mm, cerebral complications, abscess, and failure to undertake surgery when indicated. Indications for surgery were hemodynamic in 46.3% of cases, embolic in 32.1%, and infectious in 64.2% (the latter a very percentage, which is different from other large series of IE). Surgery was performed emergently in 6.7%, urgently in 24.8%, beyond the 1st week in 32% and electively in 36.5%. Having an indication for surgery and not performing surgery was the group with the highest mortality in the study and figured as the take home message. Importantly, the main reason for not performing surgery was death before surgery (53%) of patients.^3^

It seems clear that referring early for surgical evaluation by a team experienced in treating endocarditis and performing surgery in a timely manner is important. The timeframe between surgical indication and operation was 2 weeks in the article by Marques et al.,^1^ only a third of the patients were operated on, and 2/3 of patients did not have surgery indicated due to significant comorbidities.^1^

In a prospective observational study on IE from multiple sites, surgical treatment for IE was performed in 733 patients, which represented 57% of all patients and 76% of patients with a surgical indication.^6^ The median age was 57 years for patients who underwent surgery, statistically different compared with 68 years for those who did not undergo surgery. Patients who underwent surgery were more likely to have new moderate or severe mitral or aortic regurgitation, valve perforation or abscess and embolization. In contrast, patients who did not undergo surgical treatment for IE were more likely to have medical comorbidities such as coronary artery disease, previous heart failure, diabetes mellitus and moderate/severe renal disease (findings on comorbidities are similar^3^) and to have infection caused by *S. aureus*. In-hospital mortality was 26% vs 14.8% and 6-month mortality 31.4% versus 17.5% among patients who did not undergo surgery compared with those who did, respectively. The reasons for lack of surgery for those who had surgical indications were having a poor prognosis regardless of treatment (33.7%), hemodynamic instability (19.8%), death before surgery (23.3%), stroke (22.7%), and sepsis (21.0%). Sepsis was the single factor associated with nonsurgical management of *S. aureus* IE compared with other microbiological causes and median STS-IE score for *S. aureus* patients was higher (32) compared with 24 in non-*S aureus* patients, with statistical significance.

In the study by Marques et al.,^1^ as expected, septic shock was associated with mortality, with an OR of 20. Sepsis remains a challenge, with very high mortality rates worldwide, especially when associated with shock.^10^ Main therapeutic measures are dealt with in the Surviving Sepsis Campaigns, of which the most recent version reinforces speediness in starting intravenous fluids, collecting blood cultures, starting appropriate antibiotics soon after this, measuring lactate, and importantly, starting vasoactive drugs readily (within 1 hour) if intravenous fluids fail to improve blood pressure and normalize lactate levels.^11^

Despite the benefits in the survival of surgery, many deaths occur after surgery, and prognostic scores for valvular surgery in IE have been debated in recent years. Mortality rates in the EUROENDO study^3^ shows that in hospital post-cardiac surgery mortality was 170/532 (32%) overall, 74/187 (39.6%) if it was prosthetic IE and 79/286 (27.6%) if native valve IE. A recent small study from our team included 154 patients operated for IE from 2006-2016; they were mostly male (66.9%), and mean age was 42.7±15 years.^12^ Rheumatic valvulopathy was present in 31.2%; the most frequently isolated microorganisms were *viridans* group streptococci (29.9%), followed by negative cultures in 26.6% of the patients. The main surgical indication was heart failure (65.6%), and in-hospital mortality was 17.5%. On multivariate analysis, variables found to be statistically significant for death were atrioventricular block, cardiogenic shock, insulin-dependent diabetes mellitus, non-HACEK Gram-negatives as the etiology of IE and inotropic use. The calculated sensitivity for this was 88.9% and specificity was 91.8%; AUC was 0.97. This was dubbed INC-Rio score, and an app for Android was created (endocarditeinc.org).

In the present study^1^ IE with negative blood cultures was associated with mortality; a publication from our group showed that, although there was no difference in mortality for blood culture positive IE and blood culture-negative IE, the latter was associated with more heart failure, which is the main factor associated with death in IE and the main reason to indicate cardiac surgery in most series.^13^

In conclusion, the manuscript by Marques et al, despite limited in its inferences due to the retrospective, single-center nature of the study, is important as it brings to the cardiologists’ attention the issue of the very high mortality associated with IE, especially in a center with no cardiac surgery. The important message is conveyed: left-sided IE is very often a surgical disease, and an endocarditis team is more expedite in recognizing and better treating this condition, especially with respect to indicating surgery, hopefully at its most appropriate moment.
